# O02 How equitable are OPAT and cOPAT services at caring for patients who inject drugs? Initial results of a national questionnaire based stocktake

**DOI:** 10.1093/jacamr/dlaf239.002

**Published:** 2026-01-05

**Authors:** Daisy Woolham, Joanne Munns, Claire Mackintosh, Bethany Davies

**Affiliations:** Brighton and Sussex Medical School, Brighton, UK; University Hospitals Sussex, UK; University Hospitals Sussex, UK; Regional Infectious Disease Unit, NHS Lothian and Centre for Homelessness and Inclusion Health, University of Edinburgh, Edinburgh, UK; Brighton and Sussex Medical School, Brighton, UK; University Hospitals Sussex, UK

## Abstract

Patients who inject drugs (PWID) experience a high burden of injecting-related bacterial infections (IRI), which can have sequelae such as bacteraemia, abscess, endocarditis and bone and joint infections. Treatment for these infections has traditionally mandated hospital admission for IV antibiotics. PWID patients often struggle with hospital admission, with high rates of self-discharge and non-completion.

In non PWID patients with similar infections, OPAT and cOPAT (complex oral antibiotics) are increasingly used to provide antimicrobial treatment outside of hospital in a community setting. However, in the UK, national OPAT guidance does not offer recommendations on whether OPAT should be offered for patients who inject drugs due to limited evidence. Large-scale trials of early oral switch for endocarditis and bone and joint infections did not include PWID, leaving a grey area. Dalbavancin, a long-acting glycopeptide is widely used in the UK off license in particular for PWID patients, and outcomes and efficacy of this approach remain under study. In other global settings such as the USA, injecting drug use is not a contra-indication to referral to OPAT, and some UK OPAT centres have shifted to more inclusive models of care including increasing the inclusivity of cOPAT services.

To date there has been no formal evaluation or stocktake of the different services and approaches of different OPAT services around the UK towards caring for PWID patients. This paper will share the initial results of a BSAC-partnered national service evaluation using an electronic questionnaire methodology to map the service provision for PWID among infection and ambulatory services and explore the attitudes and perceptions of clinicians with regarding to improving inclusivity of care.

